# Supplementary material for: Occupational exposures and mitigation strategies among homeless shelter workers at risk of COVID-19
Source: PLoS One. 2021 Nov 1;16(11):e0253108. doi: 10.1371/journal.pone.0253108 (PMC8559982; doi:10.1371/journal.pone.0253108)
Supplement: S1 Data — (PDF) [file pone.0253108.s001.pdf]

Variable	Question	Field Type	Values, Labels
record_id	Record ID	text	
shelter_network	Shelter Network	number	
d_cur_smoker	Do you currently smoke tobacco?	dropdown	0, No|1, Yes
d_past_smoker	Are you a past smoker?	dropdown	0, No|1, Yes
d_cur_vape	Do you currently vape/smoke electronic cigarettes?	dropdown	0, No|1, Yes
d_household_num	How many people live in your household, including yourself?	text	
d_flu_vax	Did you get the flu vaccine (flu shot) this year (i.e., between Oct 2019 to present)	dropdown	0, No|1, Yes
d_underlying_ill___0	Do you have any of the following underlying illnesses? (choice=Chronic lung disease (asthma/emphysema/COPD))	checkbox	0, Unchecked|1, Checked
d_underlying_ill___1	Do you have any of the following underlying illnesses? (choice=Diabetes)	checkbox	0, Unchecked|1, Checked
d_underlying_ill___2	Do you have any of the following underlying illnesses? (choice=Heart disease)	checkbox	0, Unchecked|1, Checked
d_underlying_ill___3	Do you have any of the following underlying illnesses? (choice=Chronic kidney disease)	checkbox	0, Unchecked|1, Checked
d_underlying_ill___4	Do you have any of the following underlying illnesses? (choice=Chronic liver disease)	checkbox	0, Unchecked|1, Checked
d_underlying_ill___5	Do you have any of the following underlying illnesses? (choice=Neurologic/neurodevelopmental/intellectual disability)	checkbox	0, Unchecked|1, Checked
d_underlying_ill___6	Do you have any of the following underlying illnesses? (choice=Other chronic diseases)	checkbox	0, Unchecked|1, Checked
d_underlying_ill___7	Do you have any of the following underlying illnesses? (choice=None)	checkbox	0, Unchecked|1, Checked
d_underlying_ill___8	Do you have any of the following underlying illnesses? (choice=High Blood Pressure)	checkbox	0, Unchecked|1, Checked
d_immuno_cond	Do you have an immunosuppressive condition (i.e., a condition that weakens your immune system such as rheumatoid	dropdown	0, No|1, Yes
d_ht_ft	What is your height?Feet:	dropdown	
d_ht_in	inches:	dropdown	
d_wt	What is your weight (pounds)?	text	
test_tested	Have you been tested for COVID-19?	dropdown	0, No|1, Yes
test_times	How many times were you tested? (Enter up to 10 tests	text	
test_1_loc	Where did you get access to testing?	dropdown	0, Workplace|1, Medical Provider|2, Other
test_1_type	Was it a blood test?	dropdown	0, No|1, Yes
test_1_symp___0	In the month before testing or two months after testing, did you have any of the following symptoms? (choice=Cough (new onset or worsening of chronic cough))	checkbox	0, Unchecked|1, Checked
test_1_symp___1	In the month before testing or two months after testing, did you have any of the following symptoms? (choice=Headache)	checkbox	0, Unchecked|1, Checked
test_1_symp___2	In the month before testing or two months after testing, did you have any of the following symptoms? (choice=Muscle pain)	checkbox	0, Unchecked|1, Checked
test_1_symp___3	In the month before testing or two months after testing, did you have any of the following symptoms? (choice=Fever/feverish)	checkbox	0, Unchecked|1, Checked
test_1_symp___4	In the month before testing or two months after testing, did you have any of the following symptoms? (choice=New loss of smell/taste)	checkbox	0, Unchecked|1, Checked
test_1_symp___5	In the month before testing or two months after testing, did you have any of the following symptoms? (choice=Chills)	checkbox	0, Unchecked|1, Checked
test_1_symp___6	In the month before testing or two months after testing, did you have any of the following symptoms? (choice=Difficulty breathing)	checkbox	0, Unchecked|1, Checked
test_1_symp___7	In the month before testing or two months after testing, did you have any of the following symptoms? (choice=Sore throat)	checkbox	0, Unchecked|1, Checked
test_1_symp___8	In the month before testing or two months after testing, did you have any of the following symptoms? (choice=Diarrhea (3 or more loose/looser than normal stools/24hr period))	checkbox	0, Unchecked|1, Checked
test_1_symp___9	In the month before testing or two months after testing, did you have any of the following symptoms? (choice=Other)	checkbox	0, Unchecked|1, Checked
test_1_symp___10	In the month before testing or two months after testing, did you have any of the following symptoms? (choice=None)	checkbox	0, Unchecked|1, Checked
test_1_symp_work	If yes, did you go to work while feeling these symptoms?	dropdown	0, No|1, Yes
test_1_symp_care	Did you seek medical care for your symptoms?	dropdown	0, No|1, Yes
test_1_days	How many days did it take to get your results?	text	
test_1_work	Did you work while waiting for results?	dropdown	0, No|1, Yes
test_1_result	Result of test	dropdown	0, Negative|1, Positive|2, Don't know
test_2_loc	Where did you get access to testing?	dropdown	0, Workplace|1, Medical Provider|2, Other
test_2_type	Was it a blood test?	dropdown	0, No|1, Yes
test_2_symp___0	In the month before testing or two months after testing, did you have any of the following symptoms? (choice=Cough (new onset or worsening of chronic cough))	checkbox	0, Unchecked|1, Checked
test_2_symp___1	In the month before testing or two months after testing, did you have any of the following symptoms? (choice=Headache)	checkbox	0, Unchecked|1, Checked
test_2_symp___2	In the month before testing or two months after testing, did you have any of the following symptoms? (choice=Muscle pain)	checkbox	0, Unchecked|1, Checked
test_2_symp___3	In the month before testing or two months after testing, did you have any of the following symptoms? (choice=Fever/feverish)	checkbox	0, Unchecked|1, Checked
test_2_symp___4	In the month before testing or two months after testing, did you have any of the following symptoms? (choice=New loss of smell/taste)	checkbox	0, Unchecked|1, Checked
test_2_symp___5	In the month before testing or two months after testing, did you have any of the following symptoms? (choice=Chills)	checkbox	0, Unchecked|1, Checked
test_2_symp___6	In the month before testing or two months after testing, did you have any of the following symptoms? (choice=Difficulty breathing)	checkbox	0, Unchecked|1, Checked
test_2_symp___7	In the month before testing or two months after testing, did you have any of the following symptoms? (choice=Sore throat)	checkbox	0, Unchecked|1, Checked
test_2_symp___8	In the month before testing or two months after testing, did you have any of the following symptoms? (choice=Diarrhea (3 or more loose/looser than normal stools/24hr period))	checkbox	0, Unchecked|1, Checked
test_2_symp___9	In the month before testing or two months after testing, did you have any of the following symptoms? (choice=Other)	checkbox	0, Unchecked|1, Checked
test_2_symp___10	In the month before testing or two months after testing, did you have any of the following symptoms? (choice=None)	checkbox	0, Unchecked|1, Checked
test_2_symp_work	If yes, did you go to work while feeling these symptoms?	dropdown	0, No|1, Yes
test_2_symp_spec	Please specify why you went to work:	text	
test_2_symp_care	Did you seek medical care for your symptoms?	dropdown	0, No|1, Yes
test_2_days	How many days did it take to get your results?	text	
test_2_work	Did you work while waiting for results?	dropdown	0, No|1, Yes
test_2_result	Result of test	dropdown	0, Negative|1, Positive|2, Don't know
test_3_loc	Where did you get access to testing?	dropdown	0, Workplace|1, Medical Provider|2, Other
test_3_type	Was it a blood test?	dropdown	0, No|1, Yes
test_3_symp___0	In the month before testing or two months after testing, did you have any of the following symptoms? (choice=Cough (new onset or worsening of chronic cough))	checkbox	0, Unchecked|1, Checked
test_3_symp___1	In the month before testing or two months after testing, did you have any of the following symptoms? (choice=Headache)	checkbox	0, Unchecked|1, Checked
test_3_symp___2	In the month before testing or two months after testing, did you have any of the following symptoms? (choice=Muscle pain)	checkbox	0, Unchecked|1, Checked
test_3_symp___3	In the month before testing or two months after testing, did you have any of the following symptoms? (choice=Fever/feverish)	checkbox	0, Unchecked|1, Checked
test_3_symp___4	In the month before testing or two months after testing, did you have any of the following symptoms? (choice=New loss of smell/taste)	checkbox	0, Unchecked|1, Checked

test_3_symp___5	In the month before testing or two months after testing, did you have any of the following symptoms? (choice=Chills)	checkbox	0, Unchecked|1, Checked
test_3_symp___6	In the month before testing or two months after testing, did you have any of the following symptoms? (choice=Difficulty breathing)	checkbox	0, Unchecked|1, Checked
test_3_symp___7	In the month before testing or two months after testing, did you have any of the following symptoms? (choice=Sore throat)	checkbox	0, Unchecked|1, Checked
test_3_symp___8	In the month before testing or two months after testing, did you have any of the following symptoms? (choice=Diarrhea (3 or more loose/looser than normal stools/24hr period))	checkbox	0, Unchecked|1, Checked
test_3_symp___9	In the month before testing or two months after testing, did you have any of the following symptoms? (choice=Other)	checkbox	0, Unchecked|1, Checked
test_3_symp___10	In the month before testing or two months after testing, did you have any of the following symptoms? (choice=None)	checkbox	0, Unchecked|1, Checked
test_3_symp_work	If yes, did you go to work while feeling these symptoms?	dropdown	0, No|1, Yes
test_3_symp_care	Did you seek medical care for your symptoms?	dropdown	0, No|1, Yes
test_3_days	How many days did it take to get your results?	text	
test_3_work	Did you work while waiting for results?	dropdown	0, No|1, Yes
test_3_result	Result of test	dropdown	0, Negative|1, Positive|2, Don't know
test_4_loc	Where did you get access to testing?	dropdown	0, Workplace|1, Medical Provider|2, Other
test_4_type	Was it a blood test?	dropdown	0, No|1, Yes
test_4_symp___0	In the month before testing or two months after testing, did you have any of the following symptoms? (choice=Cough (new onset or worsening of chronic cough))	checkbox	0, Unchecked|1, Checked
test_4_symp___1	In the month before testing or two months after testing, did you have any of the following symptoms? (choice=Headache)	checkbox	0, Unchecked|1, Checked
test_4_symp___2	In the month before testing or two months after testing, did you have any of the following symptoms? (choice=Muscle pain)	checkbox	0, Unchecked|1, Checked
test_4_symp___3	In the month before testing or two months after testing, did you have any of the following symptoms? (choice=Fever/feverish)	checkbox	0, Unchecked|1, Checked
test_4_symp___4	In the month before testing or two months after testing, did you have any of the following symptoms? (choice=New loss of smell/taste)	checkbox	0, Unchecked|1, Checked
test_4_symp___5	In the month before testing or two months after testing, did you have any of the following symptoms? (choice=Chills)	checkbox	0, Unchecked|1, Checked
test_4_symp___6	In the month before testing or two months after testing, did you have any of the following symptoms? (choice=Difficulty breathing)	checkbox	0, Unchecked|1, Checked
test_4_symp___7	In the month before testing or two months after testing, did you have any of the following symptoms? (choice=Sore throat)	checkbox	0, Unchecked|1, Checked
test_4_symp___8	In the month before testing or two months after testing, did you have any of the following symptoms? (choice=Diarrhea (3 or more loose/looser than normal stools/24hr period))	checkbox	0, Unchecked|1, Checked
test_4_symp___9	In the month before testing or two months after testing, did you have any of the following symptoms? (choice=Other)	checkbox	0, Unchecked|1, Checked
test_4_symp___10	In the month before testing or two months after testing, did you have any of the following symptoms? (choice=None)	checkbox	0, Unchecked|1, Checked
test_4_symp_work	If yes, did you go to work while feeling these symptoms?	dropdown	0, No|1, Yes
test_4_symp_care	Did you seek medical care for your symptoms?	dropdown	0, No|1, Yes
test_4_days	How many days did it take to get your results?	text	
test_4_work	Did you work while waiting for results?	dropdown	0, No|1, Yes
test_4_result	Result of test	dropdown	0, Negative|1, Positive|2, Don't know
test_5_loc	Where did you get access to testing?	dropdown	0, Workplace|1, Medical Provider|2, Other
test_5_type	Was it a blood test?	dropdown	0, No|1, Yes
test_5_symp___0	In the month before testing or two months after testing, did you have any of the following symptoms? (choice=Cough (new onset or worsening of chronic cough))	checkbox	0, Unchecked|1, Checked
test_5_symp___1	In the month before testing or two months after testing, did you have any of the following symptoms? (choice=Headache)	checkbox	0, Unchecked|1, Checked
test_5_symp___2	In the month before testing or two months after testing, did you have any of the following symptoms? (choice=Muscle pain)	checkbox	0, Unchecked|1, Checked
test_5_symp___3	In the month before testing or two months after testing, did you have any of the following symptoms? (choice=Fever/feverish)	checkbox	0, Unchecked|1, Checked
test_5_symp___4	In the month before testing or two months after testing, did you have any of the following symptoms? (choice=New loss of smell/taste)	checkbox	0, Unchecked|1, Checked
test_5_symp___5	In the month before testing or two months after testing, did you have any of the following symptoms? (choice=Chills)	checkbox	0, Unchecked|1, Checked
test_5_symp___6	In the month before testing or two months after testing, did you have any of the following symptoms? (choice=Difficulty breathing)	checkbox	0, Unchecked|1, Checked
test_5_symp___7	In the month before testing or two months after testing, did you have any of the following symptoms? (choice=Sore throat)	checkbox	0, Unchecked|1, Checked
test_5_symp___8	In the month before testing or two months after testing, did you have any of the following symptoms? (choice=Diarrhea (3 or more loose/looser than normal stools/24hr period))	checkbox	0, Unchecked|1, Checked
test_5_symp___9	In the month before testing or two months after testing, did you have any of the following symptoms? (choice=Other)	checkbox	0, Unchecked|1, Checked
test_5_symp___10	In the month before testing or two months after testing, did you have any of the following symptoms? (choice=None)	checkbox	0, Unchecked|1, Checked
test_5_symp_work	If yes, did you go to work while feeling these symptoms?	dropdown	0, No|1, Yes
test_5_symp_care	Did you seek medical care for your symptoms?	dropdown	0, No|1, Yes
test_5_days	How many days did it take to get your results?	text	
test_5_work	Did you work while waiting for results?	dropdown	0, No|1, Yes
test_5_result	Result of test	dropdown	0, Negative|1, Positive|2, Don't know
test_6_loc	Where did you get access to testing?	dropdown	0, Workplace|1, Medical Provider|2, Other
test_6_type	Was it a blood test?	dropdown	0, No|1, Yes
test_6_symp___0	In the month before testing or two months after testing, did you have any of the following symptoms? (choice=Cough (new onset or worsening of chronic cough))	checkbox	0, Unchecked|1, Checked
test_6_symp___1	In the month before testing or two months after testing, did you have any of the following symptoms? (choice=Headache)	checkbox	0, Unchecked|1, Checked
test_6_symp___2	In the month before testing or two months after testing, did you have any of the following symptoms? (choice=Muscle pain)	checkbox	0, Unchecked|1, Checked
test_6_symp___3	In the month before testing or two months after testing, did you have any of the following symptoms? (choice=Fever/feverish)	checkbox	0, Unchecked|1, Checked
test_6_symp___4	In the month before testing or two months after testing, did you have any of the following symptoms? (choice=New loss of smell/taste)	checkbox	0, Unchecked|1, Checked
test_6_symp___5	In the month before testing or two months after testing, did you have any of the following symptoms? (choice=Chills)	checkbox	0, Unchecked|1, Checked
test_6_symp___6	In the month before testing or two months after testing, did you have any of the following symptoms? (choice=Difficulty breathing)	checkbox	0, Unchecked|1, Checked
test_6_symp___7	In the month before testing or two months after testing, did you have any of the following symptoms? (choice=Sore throat)	checkbox	0, Unchecked|1, Checked

test_6_symp___8	In the month before testing or two months after testing, did you have any of the following symptoms? (choice=Diarrhea (3 or more loose/looser than normal stools/24hr period))	checkbox	0, Unchecked|1, Checked
test_6_symp___9	In the month before testing or two months after testing, did you have any of the following symptoms? (choice=Other)	checkbox	0, Unchecked|1, Checked
test_6_symp___10	In the month before testing or two months after testing, did you have any of the following symptoms? (choice=None)	checkbox	0, Unchecked|1, Checked
test_6_symp_work	If yes, did you go to work while feeling these symptoms?	dropdown	0, No|1, Yes
test_6_symp_care	Did you seek medical care for your symptoms?	dropdown	0, No|1, Yes
test_6_days	How many days did it take to get your results?	text	
test_6_work	Did you work while waiting for results?	dropdown	0, No|1, Yes
test_6_result	Result of test	dropdown	0, Negative|1, Positive|2, Don't know
test_7_loc	Where did you get access to testing?	dropdown	0, Workplace|1, Medical Provider|2, Other
test_7_type	Was it a blood test?	dropdown	0, No|1, Yes
test_7_symp___0	In the month before testing or two months after testing, did you have any of the following symptoms? (choice=Cough (new onset or worsening of chronic cough))	checkbox	0, Unchecked|1, Checked
test_7_symp___1	In the month before testing or two months after testing, did you have any of the following symptoms? (choice=Headache)	checkbox	0, Unchecked|1, Checked
test_7_symp___2	In the month before testing or two months after testing, did you have any of the following symptoms? (choice=Muscle pain)	checkbox	0, Unchecked|1, Checked
test_7_symp___3	In the month before testing or two months after testing, did you have any of the following symptoms? (choice=Fever/feverish)	checkbox	0, Unchecked|1, Checked
test_7_symp___4	In the month before testing or two months after testing, did you have any of the following symptoms? (choice=New loss of smell/taste)	checkbox	0, Unchecked|1, Checked
test_7_symp___5	In the month before testing or two months after testing, did you have any of the following symptoms? (choice=Chills)	checkbox	0, Unchecked|1, Checked
test_7_symp___6	In the month before testing or two months after testing, did you have any of the following symptoms? (choice=Difficulty breathing)	checkbox	0, Unchecked|1, Checked
test_7_symp___7	In the month before testing or two months after testing, did you have any of the following symptoms? (choice=Sore throat)	checkbox	0, Unchecked|1, Checked
test_7_symp___8	In the month before testing or two months after testing, did you have any of the following symptoms? (choice=Diarrhea (3 or more loose/looser than normal stools/24hr period))	checkbox	0, Unchecked|1, Checked
test_7_symp___9	In the month before testing or two months after testing, did you have any of the following symptoms? (choice=Other)	checkbox	0, Unchecked|1, Checked
test_7_symp___10	In the month before testing or two months after testing, did you have any of the following symptoms? (choice=None)	checkbox	0, Unchecked|1, Checked
test_7_symp_work	If yes, did you go to work while feeling these symptoms?	dropdown	0, No|1, Yes
test_7_symp_care	Did you seek medical care for your symptoms?	dropdown	0, No|1, Yes
test_7_days	How many days did it take to get your results?	text	
test_7_work	Did you work while waiting for results?	dropdown	0, No|1, Yes
test_7_result	Result of test	dropdown	0, Negative|1, Positive|2, Don't know
test_8_loc	Where did you get access to testing?	dropdown	0, Workplace|1, Medical Provider|2, Other
test_8_type	Was it a blood test?	dropdown	0, No|1, Yes
test_8_symp___0	In the month before testing or two months after testing, did you have any of the following symptoms? (choice=Cough (new onset or worsening of chronic cough))	checkbox	0, Unchecked|1, Checked
test_8_symp___1	In the month before testing or two months after testing, did you have any of the following symptoms? (choice=Headache)	checkbox	0, Unchecked|1, Checked
test_8_symp___2	In the month before testing or two months after testing, did you have any of the following symptoms? (choice=Muscle pain)	checkbox	0, Unchecked|1, Checked
test_8_symp___3	In the month before testing or two months after testing, did you have any of the following symptoms? (choice=Fever/feverish)	checkbox	0, Unchecked|1, Checked
test_8_symp___4	In the month before testing or two months after testing, did you have any of the following symptoms? (choice=New loss of smell/taste)	checkbox	0, Unchecked|1, Checked
test_8_symp___5	In the month before testing or two months after testing, did you have any of the following symptoms? (choice=Chills)	checkbox	0, Unchecked|1, Checked
test_8_symp___6	In the month before testing or two months after testing, did you have any of the following symptoms? (choice=Difficulty breathing)	checkbox	0, Unchecked|1, Checked
test_8_symp___7	In the month before testing or two months after testing, did you have any of the following symptoms? (choice=Sore throat)	checkbox	0, Unchecked|1, Checked
test_8_symp___8	In the month before testing or two months after testing, did you have any of the following symptoms? (choice=Diarrhea (3 or more loose/looser than normal stools/24hr period))	checkbox	0, Unchecked|1, Checked
test_8_symp___9	In the month before testing or two months after testing, did you have any of the following symptoms? (choice=Other)	checkbox	0, Unchecked|1, Checked
test_8_symp___10	In the month before testing or two months after testing, did you have any of the following symptoms? (choice=None)	checkbox	0, Unchecked|1, Checked
test_8_symp_work	If yes, did you go to work while feeling these symptoms?	dropdown	0, No|1, Yes
test_8_symp_care	Did you seek medical care for your symptoms?	dropdown	0, No|1, Yes
test_8_days	How many days did it take to get your results?	text	
test_8_work	Did you work while waiting for results?	dropdown	0, No|1, Yes
test_8_result	Result of test	dropdown	0, Negative|1, Positive|2, Don't know
test_9_loc	Where did you get access to testing?	dropdown	0, Workplace|1, Medical Provider|2, Other
test_9_type	Was it a blood test?	dropdown	0, No|1, Yes
test_9_symp___0	In the month before testing or two months after testing, did you have any of the following symptoms? (choice=Cough (new onset or worsening of chronic cough))	checkbox	0, Unchecked|1, Checked
test_9_symp___1	In the month before testing or two months after testing, did you have any of the following symptoms? (choice=Headache)	checkbox	0, Unchecked|1, Checked
test_9_symp___2	In the month before testing or two months after testing, did you have any of the following symptoms? (choice=Muscle pain)	checkbox	0, Unchecked|1, Checked
test_9_symp___3	In the month before testing or two months after testing, did you have any of the following symptoms? (choice=Fever/feverish)	checkbox	0, Unchecked|1, Checked
test_9_symp___4	In the month before testing or two months after testing, did you have any of the following symptoms? (choice=New loss of smell/taste)	checkbox	0, Unchecked|1, Checked
test_9_symp___5	In the month before testing or two months after testing, did you have any of the following symptoms? (choice=Chills)	checkbox	0, Unchecked|1, Checked
test_9_symp___6	In the month before testing or two months after testing, did you have any of the following symptoms? (choice=Difficulty breathing)	checkbox	0, Unchecked|1, Checked
test_9_symp___7	In the month before testing or two months after testing, did you have any of the following symptoms? (choice=Sore throat)	checkbox	0, Unchecked|1, Checked
test_9_symp___8	In the month before testing or two months after testing, did you have any of the following symptoms? (choice=Diarrhea (3 or more loose/looser than normal stools/24hr period))	checkbox	0, Unchecked|1, Checked
test_9_symp___9	In the month before testing or two months after testing, did you have any of the following symptoms? (choice=Other)	checkbox	0, Unchecked|1, Checked
test_9_symp___10	In the month before testing or two months after testing, did you have any of the following symptoms? (choice=None)	checkbox	0, Unchecked|1, Checked
test_9_symp_work	If yes, did you go to work while feeling these symptoms?	dropdown	0, No|1, Yes

test_9_symp_care	Did you seek medical care for your symptoms?	dropdown	0, No|1, Yes
test_9_days	How many days did it take to get your results?	text	
test_9_work	Did you work while waiting for results?	dropdown	0, No|1, Yes
test_9_result	Result of test	dropdown	0, Negative|1, Positive|2, Don't know
test_10_loc	Where did you get access to testing?	dropdown	0, Workplace|1, Medical Provider|2, Other
test_10_type	Was it a blood test?	dropdown	0, No|1, Yes
test_10_symp___0	In the month before testing or two months after testing, did you have any of the following symptoms? (choice=Cough (new onset or worsening of chronic cough))	checkbox	0, Unchecked|1, Checked
test_10_symp___1	In the month before testing or two months after testing, did you have any of the following symptoms? (choice=Headache)	checkbox	0, Unchecked|1, Checked
test_10_symp___2	In the month before testing or two months after testing, did you have any of the following symptoms? (choice=Muscle pain)	checkbox	0, Unchecked|1, Checked
test_10_symp___3	In the month before testing or two months after testing, did you have any of the following symptoms? (choice=Fever/feverish)	checkbox	0, Unchecked|1, Checked
test_10_symp___4	In the month before testing or two months after testing, did you have any of the following symptoms? (choice=New loss of smell/taste)	checkbox	0, Unchecked|1, Checked
test_10_symp___5	In the month before testing or two months after testing, did you have any of the following symptoms? (choice=Chills)	checkbox	0, Unchecked|1, Checked
test_10_symp___6	In the month before testing or two months after testing, did you have any of the following symptoms? (choice=Difficulty breathing)	checkbox	0, Unchecked|1, Checked
test_10_symp___7	In the month before testing or two months after testing, did you have any of the following symptoms? (choice=Sore throat)	checkbox	0, Unchecked|1, Checked
test_10_symp___8	In the month before testing or two months after testing, did you have any of the following symptoms? (choice=Diarrhea (3 or more loose/looser than normal stools/24hr period))	checkbox	0, Unchecked|1, Checked
test_10_symp___9	In the month before testing or two months after testing, did you have any of the following symptoms? (choice=Other)	checkbox	0, Unchecked|1, Checked
test_10_symp___10	In the month before testing or two months after testing, did you have any of the following symptoms? (choice=None)	checkbox	0, Unchecked|1, Checked
test_10_symp_work	If yes, did you go to work while feeling these symptoms?	dropdown	0, No|1, Yes
test_10_symp_care	Did you seek medical care for your symptoms?	dropdown	0, No|1, Yes
test_10_days	How many days did it take to get your results?	text	
test_10_work	Did you work while waiting for results?	dropdown	0, No|1, Yes
test_10_result	Result of test	dropdown	0, Negative|1, Positive|2, Don't know
test_pos	If tested positive, how were you told?	radio	0, My supervisor|1, I called|2, Health department|3, My doctor|4, Don't remember|5, Other
test_infect_loc	Where do you think you were infected?	radio	0, Home|1, Work|2, Community|3, I don't know|4, Skip
test_pos_instruc	What did management tell you to do after you were diagnosed?	radio	0, Stay home and isolate|1, Continue to work|2, No instruction provided|3, Other
job_cat	Job Category	string	"Administrative" | "Client engagement"
we_hours	On average, how many hours do you work in a week at this shelter?	text	
we_years	How long have you worked at this shelter? Years:	text	
we_months	months:	dropdown	
we_sick_leave	Do you have paid sick leave at this job?	dropdown	0, No|1, Yes
we_other_job	Do you have another job besides this one?	dropdown	0, No|1, Yes
we_clothcov	Did you wear a reusable face covering (for example, made of cloth) at work?	radio	0, All of the time|1, Most of the time|2, Sometimes|3, Rarely/never
we_mask	Did you wear a disposable mask at work?	radio	0, All of the time|1, Most of the time|2, Sometimes|3, Rarely/never
we_resitems	Did you touch residents' belongings or shared items (e.g., mats, linens, laundry)?	dropdown	0, No|1, Yes
we_resitems_gloves	If yes, did you wear gloves while touching residents' belongings and shared items?	dropdown	0, No|1, Yes
we_contactres	Did you have close contact (less than 6 feet for more than 15 minutes at a time) or direct physical contact (e.g., touching) with residents?	dropdown	0, No|1, Yes
we_ccres_avg	On average, how often were you in close contact (less than 6 feet for more than 15 minutes at a time) with residents?	radio	0, Never|1, Rarely|2, A few times a month|3, A few times a week|4, A few times a day
we_clothcov_ccres	During those close contacts (less than 6 feet for more than 15 minutes at a time), how often did you wear a reusable face covering?	radio	0, All of the time|1, Most of the time|2, Sometimes|3, Rarely/never|4, Don't remember
we_mask_ccres	During those close contacts (less than 6 feet for more than 15 minutes at a time), how often did you wear a disposable mask?	radio	0, All of the time|1, Most of the time|2, Sometimes|3, Rarely/never|4, Don't remember
we_clothcov_ccres2	During those close contacts (less than 6 feet for more than 15 minutes at a time), how often did the residents wear a reusable face covering?	radio	0, All of the time|1, Most of the time|2, Sometimes|3, Rarely/never|4, Don't remember
we_mask_ccres2	During those close contacts (less than 6 feet for more than 15 minutes at a time), how often did the residents wear a disposable mask?	radio	0, All of the time|1, Most of the time|2, Sometimes|3, Rarely/never|4, Don't remember
we_dirphys_res	On average, how often were you in direct physical contact (e.g., touching) with residents?	radio	0, Never|1, Rarely|2, A few times a month|3, A few times a week,|4, A few times a day
we_dirphys_gloves	During those direct physical contacts (e.g., touching), how often did you wear gloves?	radio	0, All of the time|1, Most of the time|2, Sometimes|3, Rarely/never|4, Don't remember
we_ccco	How often were you in close contact (less than 6 feet for more than 15 minutes at a time) with your co-workers?	radio	0, Never|1, Rarely|2, A few times a month|3, A few times a week|4, A few times a day
we_clothcov_ccco	Did you wear facial coverings when in close contact (less than 6 feet for more than 15 minutes at a time) with your co	dropdown	0, No|1, Yes
c19_cc	Were you in close contact (less than 6 feet for more than 15 minutes at a time) with a person who had COVID-19?	dropdown	0, No|1, Yes|2, Don't know
c19_cc_exp___0	Where were you in contact with a person with COVID-19 (select all that apply)? (choice=Home)	checkbox	0, Unchecked|1, Checked
c19_cc_exp___1	Where were you in contact with a person with COVID-19 (select all that apply)? (choice=Work)	checkbox	0, Unchecked|1, Checked
c19_cc_exp___2	Where were you in contact with a person with COVID-19 (select all that apply)? (choice=Community)	checkbox	0, Unchecked|1, Checked
c19_cc_exp___3	Where were you in contact with a person with COVID-19 (select all that apply)? (choice=I don't know	checkbox	0, Unchecked|1, Checked
c19_cc_mask	Did you wear a disposable mask when interacting with the person who had COVID-19?	radio	0, Never|1, Sometimes|2, Most times|3, Always|4, Don't remember
c19_cc_ppe___0	Did you use any other PPE (personal protective equipment) when interacting with the person who had COVID-19 (select all that apply)? (choice=Gloves)	checkbox	0, Unchecked|1, Checked
c19_cc_ppe___1	Did you use any other PPE (personal protective equipment) when interacting with the person who had COVID-19 (select all that apply)? (choice=Gown)	checkbox	0, Unchecked|1, Checked
c19_cc_ppe___2	Did you use any other PPE (personal protective equipment) when interacting with the person who had COVID-19 (select all that apply)? (choice=Respirator (N-95))	checkbox	0, Unchecked|1, Checked
c19_cc_ppe___3	Did you use any other PPE (personal protective equipment) when interacting with the person who had COVID-19 (select all that apply)? (choice=None)	checkbox	0, Unchecked|1, Checked
c19_cc_ppe___4	Did you use any other PPE (personal protective equipment) when interacting with the person who had COVID-19 (select all that apply)? (choice=Other)	checkbox	0, Unchecked|1, Checked
c19_cc_ppe_other	Describe "other":	text	
c19_infect	Have you felt worried about being infected with COVID-19 because of your job?	dropdown	0, No|1, Yes|2, Skip
c19_infect_quit	Did you think of quitting your job because you were worried?	dropdown	0, No|1, Yes|2, Skip
home_famsup	Is your family supportive of your work?	dropdown	0, No|1, Yes|2, Unknown|3, Skip
home_risk_you	Did your family/friends feel you were at increased risk of being infected with COVID-19 due to the nature of your work?	dropdown	0, No|1, Yes
home_risk_fam	Did you feel they (your friends/family) were at increased risk of being infected with COVID-19 due to the nature of your	dropdown	0, No|1, Yes

ipc_educ	Do you have any formal health education (i.e., medical school, nursing school, medical assistant school, behavioral	dropdown	0, No|1, Yes|2, Other
ipc_clean	Did you perform cleaning activities in your normal job?	dropdown	0, No|1, Yes
ipc_clean_c19	Were you trained on how to clean an area after a resident diagnosed with COVID-19 leaves the shelter?	dropdown	0, No|1, Yes
ipc_resp___0	What did your facility do in response to the COVID-19 outbreak (select all that apply)? (choice=Increased handwashing	checkbox	0, Unchecked|1, Checked
ipc_resp___1	What did your facility do in response to the COVID-19 outbreak (select all that apply)? (choice=Safe distancing (more than	checkbox	0, Unchecked|1, Checked
ipc_resp___2	What did your facility do in response to the COVID-19 outbreak (select all that apply)? (choice=Cloth face coverings for	checkbox	0, Unchecked|1, Checked
ipc_resp___3	What did your facility do in response to the COVID-19 outbreak (select all that apply)? (choice=Provision of PPE (personal protective equipment, such as gloves, gowns, disposable mask) for staff)	checkbox	0, Unchecked|1, Checked
ipc_resp___4	What did your facility do in response to the COVID-19 outbreak (select all that apply)? (choice=Nothing	checkbox	0, Unchecked|1, Checked
ipc_act_agree	Do you agree with how your organization responded to the outbreak?	dropdown	0, No|1, Yes|2, Don't know|3, Skip
