# Supplementary material for: Occupational exposures and mitigation strategies among homeless shelter workers at risk of COVID-19
Source: PLoS One. 2021 Nov 1;16(11):e0253108. doi: 10.1371/journal.pone.0253108 (PMC8559982; doi:10.1371/journal.pone.0253108)
Supplement: S1 File — (PDF) [file pone.0253108.s003.pdf]

Homeless Shelter Worker Survey

Thank you for participating in this survey. By providing information on your experiences prior to being tested for COVID-19, you will help inform ways to protect staff and clients from Coronavirus Disease 2019 (COVID-19) at homeless shelters throughout the United States.

The results of this survey are confidential. Your name and affiliation will not be recorded. When survey results are reported they will be aggregated and presented at a county, state, or continuum of care level.

The survey will take less than 15 minutes. If you are not able to complete the survey at this time you can save it and return to it later.

Today's date for calculations (hidden) __________________________________

What is the location of the shelter you work in most often (city, state)? __________________________________

What is the name of the shelter? __________________________________

What is your age (years)? __________________________________

What was your sex at birth?
- ◯ Male
- ◯ Female

Do you think of yourself as (select one):
- ◯ Male
- ◯ Female
- ◯ Transgender man/trans man/female-to-male (FTM)
- ◯ Transgender woman/trans woman/male-to-female (MTF)
- ◯ Genderqueer/gender nonconforming neither exclusively male nor female

---

What is your race (select all that apply):
- ☐ White
- ☐ Asian
- ☐ Black
- ☐ Native Hawaiian
- ☐ Pacific Islander
- ☐ American Indian/Alaskan Native
- ☐ Other
- ☐ Skip

Describe "other": __________________________________

What is your ethnicity?
- ◯ Not Hispanic/Latino
- ◯ Hispanic/Latino
- ◯ Skip

Do you currently smoke tobacco?
- ◯ No
- ◯ Yes

Are you a past smoker?
- ◯ No
- ◯ Yes

Do you currently vape/smoke electronic cigarettes?	◯ No<br>◯ Yes
How many people live in your household, including yourself?	____________________
Did you get the flu vaccine (flu shot) this year (i.e., between Oct 2019 to present)?	◯ No<br>◯ Yes
Do you have any of the following underlying illnesses?	☐ Chronic lung disease (asthma/emphysema/COPD)<br>☐ Diabetes<br>☐ Heart disease<br>☐ Chronic kidney disease<br>☐ Chronic liver disease<br>☐ Neurologic/neurodevelopmental/intellectual disability<br>☐ Other chronic diseases<br>☐ None
Describe "other":	____________________
Do you have an immunosuppressive condition (i.e., a condition that weakens your immune system such as rheumatoid arthritis or HIV) ?	◯ No<br>◯ Yes
Please specify:	____________________
What is your height?<br><br>Feet:	◯ 3<br>◯ 4<br>◯ 5<br>◯ 6<br>◯ 7<br>◯ 8
inches:	◯ 0<br>◯ 1<br>◯ 2<br>◯ 3<br>◯ 4<br>◯ 5<br>◯ 6<br>◯ 7<br>◯ 8<br>◯ 9<br>◯ 10<br>◯ 11<br>◯ 12
What is your weight (pounds)?	____________________

TESTING AND DIAGNOSIS

Have you been tested for COVID-19?
- ◯ No
- ◯ Yes

How many times were you tested? (Enter up to 10 tests)
____

Test 1

Date of test (best guess)
____

Date of test entered is in the future, please re-enter the date before continuing.

Where did you get access to testing?
- ◯ Workplace
- ◯ Medical Provider
- ◯ Other

Describe "other":
____

Was it a blood test?
- ◯ No
- ◯ Yes

In the month before testing or two months after testing, did you have any of the following symptoms?
- ☐ Cough (new onset or worsening of chronic cough)
- ☐ Headache
- ☐ Muscle pain
- ☐ Fever/feverish
- ☐ New loss of smell/taste
- ☐ Chills
- ☐ Difficulty breathing
- ☐ Sore throat
- ☐ Diarrhea (3 or more loose/looser than normal stools/24hr period)
- ☐ Other
- ☐ None

Describe "other":
____

If yes, did you go to work while feeling these symptoms?
- ◯ No
- ◯ Yes

Please specify why you went to work:
____

Did you seek medical care for your symptoms?
- ◯ No
- ◯ Yes

If yes, where?
- ◯ Primary care
- ◯ Urgent care
- ◯ Emergency department
- ◯ Other

Describe "other":
____

How many days did it take to get your results?
____

Did you work while waiting for results?	◯ No<br>◯ Yes
Result of test	◯ Negative<br>◯ Positive<br>◯ Don't know

TESTING AND DIAGNOSIS: CONTINUED

If tested positive, how were you told?	◯ My supervisor<br>◯ I called<br>◯ Health department<br>◯ My doctor<br>◯ Don't remember<br>◯ Other
Describe "other":	__________________________________
Where do you think you were infected?	◯ Home<br>◯ Work<br>◯ Community<br>◯ I don't know<br>◯ Skip
If at work, what do you think were the activities that might have led you to become infected?	__________________________________
How do you think you were infected?	__________________________________
What did management tell you to do after you were diagnosed?	◯ Stay home and isolate<br>◯ Continue to work<br>◯ No instruction provided<br>◯ Other
Describe "other":	__________________________________

WORK ENVIRONMENT: The questions in this section refer to the time period between February 2020 and now.

What is your primary job title?	◯ Site manager<br>◯ Floor monitor<br>◯ Janitor<br>◯ Cook<br>◯ Lead staff<br>◯ Case manager<br>◯ Supervisor<br>◯ Driver<br>◯ Maintenance<br>◯ Other
Describe "other":	__________________________________

Please describe your main job duties (what you do on a typical day):	__________________________________
On average, how many hours do you work in a week at this shelter?	__________________________
How long have you worked at this shelter?<br><br>Years:	__________________________
months:	◯ 0<br>◯ 1<br>◯ 2<br>◯ 3<br>◯ 4<br>◯ 5<br>◯ 6<br>◯ 7<br>◯ 8<br>◯ 9<br>◯ 10<br>◯ 11<br>◯ 12
Do you have paid sick leave at this job?	◯ No<br>◯ Yes
Do you have another job besides this one?	◯ No<br>◯ Yes
For your other job, what is your occupation (e.g., registered nurse, janitor, cashier, auto mechanic)?	__________________________
For your other job, what is the industry (e.g., nursing home, elementary school, restaurant)?	__________________________
If your other job is at a shelter, what is the shelter name?	__________________________
Did you wear a reusable face covering (for example, made of cloth) at work?	◯ All of the time<br>◯ Most of the time<br>◯ Sometimes<br>◯ Rarely/never
Did you wear a disposable mask at work?	◯ All of the time<br>◯ Most of the time<br>◯ Sometimes<br>◯ Rarely/never
Did you touch residents' belongings or shared items (e.g., mats, linens, laundry)?	◯ No<br>◯ Yes
If yes, did you wear gloves while touching residents' belongings and shared items?	◯ No<br>◯ Yes

Did you have close contact (less than 6 feet for more than 15 minutes at a time) or direct physical contact (e.g., touching) with residents?	◯ No<br>◯ Yes
On average, how often were you in close contact (less than 6 feet for more than 15 minutes at a time) with residents?	◯ Never<br>◯ Rarely<br>◯ A few times a month<br>◯ A few times a week<br>◯ A few times a day
During those close contacts (less than 6 feet for more than 15 minutes at a time), how often did you wear a reusable face covering?	◯ All of the time<br>◯ Most of the time<br>◯ Sometimes<br>◯ Rarely/never<br>◯ Don't remember
During those close contacts (less than 6 feet for more than 15 minutes at a time), how often did you wear a disposable mask?	◯ All of the time<br>◯ Most of the time<br>◯ Sometimes<br>◯ Rarely/never<br>◯ Don't remember
During those close contacts (less than 6 feet for more than 15 minutes at a time), how often did the residents wear a reusable face covering?	◯ All of the time<br>◯ Most of the time<br>◯ Sometimes<br>◯ Rarely/never<br>◯ Don't remember
During those close contacts (less than 6 feet for more than 15 minutes at a time), how often did the residents wear a disposable mask?	◯ All of the time<br>◯ Most of the time<br>◯ Sometimes<br>◯ Rarely/never<br>◯ Don't remember
On average, how often were you in direct physical contact (e.g., touching) with residents?	◯ Never<br>◯ Rarely<br>◯ A few times a month<br>◯ A few times a week,<br>◯ A few times a day
During those direct physical contacts (e.g., touching), how often did you wear gloves?	◯ All of the time<br>◯ Most of the time<br>◯ Sometimes<br>◯ Rarely/never<br>◯ Don't remember
How often were you in close contact (less than 6 feet for more than 15 minutes at a time) with your co-workers?	◯ Never<br>◯ Rarely<br>◯ A few times a month<br>◯ A few times a week<br>◯ A few times a day
Did you wear facial coverings when in close contact (less than 6 feet for more than 15 minutes at a time) with your co-workers?	◯ No<br>◯ Yes

COVID-19 EXPERIENCE: The questions in this section refer to the time period between February 2020 and now.

Were you in close contact (less than 6 feet for more than 15 minutes at a time) with a person who had COVID-19?	○ No<br>○ Yes<br>○ Don't know
Where were you in contact with a person with COVID-19 (select all that apply)?	☐ Home<br>☐ Work<br>☐ Community<br>☐ I don't know
If at work, was that person a (select all that apply):	☐ Co-worker<br>☐ Resident<br>☐ Other
Describe "other":	__________________________
Did you wear a disposable mask when interacting with the person who had COVID-19?	○ Never<br>○ Sometimes<br>○ Most times<br>○ Always<br>○ Don't remember
Did you use any other PPE (personal protective equipment) when interacting with the person who had COVID-19 (select all that apply)?	☐ Gloves<br>☐ Gown<br>☐ Respirator (N-95)<br>☐ None<br>☐ Other
Describe "other":	__________________________
Who informed you that you had been in direct contact with a person who had COVID-19? Type NA if you were not informed.	__________________________
When were your informed, in relation to your shift?	○ Before shift<br>○ During shift<br>○ After shift
Have you felt worried about being infected with COVID-19 because of your job?	○ No<br>○ Yes<br>○ Skip
What made you worried you might get infected?	__________________________
Did you think of quitting your job because you were worried?	○ No<br>○ Yes<br>○ Skip

HOME LIFE AND FAMILY SITUATION:

Is your family supportive of your work?
- ◯ No
- ◯ Yes
- ◯ Unknown
- ◯ Skip

Did your family/friends feel you were at increased risk of being infected with COVID-19 due to the nature of your work?
- ◯ No
- ◯ Yes

Please explain. ______________________________

Did you feel they (your friends/family) were at increased risk of being infected with COVID-19 due to the nature of your work?
- ◯ No
- ◯ Yes

Have you been isolating yourself from the rest of your family, whether or not you tested positive?
- ◯ No
- ◯ Yes

Please explain. ______________________________

Did your hygiene routine at home change?
- ◯ No
- ◯ Yes

Please explain. ______________________________

INFECTION PREVENTION AND CONTROL:

Do you have any formal health education (i.e., medical school, nursing school, medical assistant school, behavioral health)?
- ◯ No
- ◯ Yes
- ◯ Other

Describe "other": ______________________________

Did you perform cleaning activities in your normal job?
- ◯ No
- ◯ Yes

Were you trained on how to clean an area after a resident diagnosed with COVID-19 leaves the shelter?
- ◯ No
- ◯ Yes

What did your facility do in response to the COVID-19 outbreak (select all that apply)?
- ☐ Increased handwashing
- ☐ Safe distancing (more than 6 ft)
- ☐ Cloth face coverings for staff or clients
- ☐ Provision of PPE (personal protective equipment, such as gloves, gowns, disposable mask) for staff
- ☐ Nothing

If yes, when did they provide PPE? ______________________________

Was training provided on how to safely take on and off PPE?
- ◯ No
- ◯ Yes

What actions did your organization recommend you take if you had been exposed to a resident with known or suspected COVID-19 (select all that apply)?	☐ Stay home and isolate<br>☐ Keep working and monitor yourself<br>☐ Work from home<br>☐ Wear a mask while working<br>☐ Work only at one facility<br>☐ New position outside of that facility<br>☐ Nothing<br>☐ Don't know<br>☐ Skip
Do you agree with how your organization responded to the outbreak?	◯ No<br>◯ Yes<br>◯ Don't know<br>◯ Skip
If no, please explain.	__________________________________
